# Ferroptosis-Related Genes Are Associated with Radioresistance and Immune Suppression in Head and Neck Cancer

**DOI:** 10.1089/gtmb.2023.0193

**Published:** 2024-03-28

**Authors:** Ping Huang, Xuejian Ning, Min Kang, RenSheng Wang

**Affiliations:** ^1^ Department of Radiation Oncology, The First Affiliated Hospital of Guangxi Medical University, Nanning, China.; ^2^ Department of Oncology, LiuZhou Traditional Chinese Medical Hospital Affiliated to Guangxi University of Chinese Medicine, Liuzhou, China.

**Keywords:** ferroptosis, head and neck cancer, radiotherapy, collagen type IV, alpha1 chain, tumor microenvironment

## Abstract

**Background:**

Ferroptosis is associated with tumor development; however, its contribution to radioresistant head and neck cancer (HNC) remains unclear. In this study, we used bioinformatics analysis and *in vitro* testing to explore ferroptosis-related genes associated with HNCs radiosensitivity.

**Materials and Methods:**

GSE9714, GSE90761, and The Cancer Genome Atlas (TCGA) datasets were searched to identify ferroptosis-related differentially expressed genes between radioresistant and radiosensitive HNCs or radiation-treated and nonradiation-treated HNCs. A protein–protein interaction analysis on identified hub genes was then performed. Receiver operating characteristic curves and Kaplan–Meier survival analysis were used to assess the diagnostic and prognostic potential of the hub genes. Cell counting kit-8, transwell assay, and flow cytometry were applied to examine the role of hub gene collagen type IV, alpha1 chain (*COL4A1*) on the proliferation, migration, invasion, and apoptosis of TU686 cells.

**Results:**

Hub genes *MMP10, MMP1, COL4A1, IFI27*, and *INHBA* showed diagnostic potential for HNC and were negatively correlated with overall survival and disease-free survival in the TCGA dataset. Also, *IL-1B, IFI27, INHBA,* and *COL4A1* mRNA levels were significantly increased in TCGA patients with advanced clinical stages or receiving radiotherapy, whereas *COL4A1, MMP10*, and *INHBA* expressions were negatively correlated with immune infiltration. Furthermore, the knockdown of *COL4A1* inhibited cell proliferation, migration, and invasion while promoting apoptosis in TU686 cells.

**Conclusion:**

Ferroptosis-related hub genes, such as *COL4A1,* are potential diagnostic and prognostic indicators as well as therapeutic targets for HNC.

## Introduction

Head and neck cancer (HNC) is the seventh most common cancer diagnosis worldwide. More than 870,000 new cases of HNC and ∼450,000 deaths are reported each year (Siegel et al., 2023). HNC is often found on the lips, oral cavity, oropharynx, hypopharynx, nasopharynx, and larynx. Despite advances in surgical techniques, chemotherapy, and radiotherapy, survival rates for HNC patients remain low, with an estimated 5-year survival rate of 65% (Siegel et al., 2020; Zolkind et al., 2021). Poor survival rates are associated with late diagnosis, considering that most patients are diagnosed with late-stage HNC (Arantes et al., 2018; Juster and Page, 2023; Rosell Ferrer et al., 2023). Also, low survival is associated with the resistance of the tumor cells to radiation-mediated death (Zhang and Yang, 2020), and immune infiltration of HNC is highly correlated with the progression of HNC and therapy response (Chang et al., 2023; Song et al., 2019). Thus, identifying immune infiltration-related biomarkers for radioresistant HNC is of utmost importance.

Ferroptosis is a newly identified iron-dependent, nonapoptotic cell death that mainly involves genetic changes in iron homeostasis and lipid peroxidation metabolism (Gao et al., 2018). Today, ferroptosis induction is considered a promising cancer treatment method (Chen et al., 2023; Shi et al., 2019 ). For example, silencing glutaredoxin 5 activates the iron-starvation response and increases intracellular free iron levels and lipid peroxidation, leading to ferroptosis in treatment-resistant HNC cells (Lee et al., 2020). Glutathione peroxidase 4 (GPX4) is a ferroptosis regulator whose overexpression promotes the proliferation of oral cancer cells (Fukuda et al., 2021). Studies have found that GPX4 inhibitors (1S, 3R)-RSL3 and ML-162 can induce ferroptosis to varying degrees in HNC cells (Shin et al., 2018). In addition, blocking the cystine transporter SLC7A11 promotes ferroptosis by targeting cystine import in HNC (Hemon et al., 2020). The above data suggest that ferroptosis-related genes have a critical role in HNC. However, little is known about their diagnostic value or biological functions in radioresistant HNC.

Bioinformatics analysis has been widely used to explore HNC diagnostic and prognostic predictors (Ju et al., 2023). Many differentially expressed genes (DEGs) have been identified between HNC and normal controls, as well as between radioresistant and radiosensitive HNCs, and characterized by gene ontology (GO) analysis, Kyoto Encyclopedia of Genes and Genomes (KEGG) pathway enrichment assay, and protein–protein interaction (PPI) network analysis (Shen et al., 2019; Yang et al., 2019; You et al., 2019). However, the relationship between DEGs and immune infiltration in HNC is still not fully understood.

In this study, we aimed to identify and characterize ferroptosis-related genes associated with HNC radiosensitivity. The relationship between the hub genes and immune infiltration was assessed. Furthermore, hub gene collagen type IV, alpha1 chain (COL4A1) was examined *in vitro* to determine its role in HNC cell proliferation, migration, invasion, and apoptosis. Our findings provide new therapeutic targets for radioresistant HNC.

## Materials and Methods

### Cell culture

Human TU686 HNC cells were purchased from BeNa Culture Collection (China) and maintained in RPMI-1640 medium (Gibco, Grand Island, NY) supplemented with 5% fetal bovine serum (FBS; Gibco) in a humified atmosphere of 5% CO_2_ at 37°C.

### Data collection and processing

The sequencing and clinicopathological data of 522 HNC patients were collected from The Cancer Genome Atlas (TCGA) database. Microarray data and clinical information of GSE9714 and GSE90761 HNC datasets were downloaded from the Gene Expression Omnibus (GEO) database (Khodarev et al., 2004). GSE9714 data were obtained using the GPL96 [HG-U133A] Affymetrix Human Genome U133A Array; GSE90761 data were obtained using the GPL22727 Illumina NextSeq 500 (mixed sample). DEGs were identified by analyzing the RNA sequencing data of the three datasets. Background corrections and data normalization were applied to the original data sets to obtain gene expression profiles.

### Identification of ferroptosis-related DEGs

DEGs were identified using the limma package (Ritchie et al., 2015). Genes with |log fold change (logFC)| > 1 and an adjusted value of *p* < 0.05 were considered DEGs. Differential mRNA expression was illustrated using a volcano plot and heatmap. Ferroptosis driver genes and ferroptosis marker genes were obtained using the online database FerrDb (www.zhounan.org/ferrdb/) (Zhou and Bao, 2020). The correlation between DEGs and ferroptosis driver/marker genes was determined using GPL96[HG-U133A] Affymetrix Human Genome U133A assay.

### Functional enrichment analysis

GO enrichment analysis was conducted using Metascape (http://metascape.org). The analysis included biological processes, molecular functions, and cellular components (Zhou et al., 2019b). GO terms with *p* < 0.01, minimum count >3, and an enrichment factor >1.5 were considered enriched GO terms. KEGG pathway analysis was performed using c2.cp.kegg. v7.2. A *q*-value <0.05 and a minimum count >3 indicated statistical significance. Gene set enrichment analysis (GSEA) was performed using 10,000 gene setup arrangements. The threshold values were *p* < 0.05 and false discovery rate (FDR) <0.25 after correction.

### PPI network

A PPI network of the DEGs was constructed using the STRING database (http://string-db.org, version 11) with a threshold value ≥0.4 (Szklarczyk et al., 2019). The PPI network was visualized using Cytoscape (Shannon et al., 2003). Hub genes were identified using cytoHubba (Chin et al., 2014).

### Immune infiltration analysis

Immune cell infiltration was analyzed using CIBERSORT (Chen et al., 2018) and TIMER2 (TIMER2.0.org) (Li et al., 2020). A Spearman correlation method was used to determine the correlation between hub genes and 24 types of immune cells.

### Receiver operating characteristic curve and survival analysis

To assess the diagnostic value of hub genes, receiver operating characteristic (ROC) curves were generated using the pROC package (Tang et al., 2017). The specificity and sensitivity of each gene were obtained. Kaplan–Meier survival analysis was performed to evaluate the prognostic value of hub genes. The correlation between hub gene expression and patients' overall survival (OS) and disease-free survival (DFS) was determined using the GEPIA online tool (Tang et al., 2017).

### Transcription factor screening

Transcription factors associated with ferroptosis-related DGEs were identified using KNOCK.TF (www.licpathway.net/KnockTF/index.html).

### Weighted gene coexpression network analysis

Gene modules related to radiotherapy were identified using the weighted gene coexpression network analysis (WGCNA) package (Langfelder and Horvath, 2008).

### Gene set variation analysis

The GSE9714 and GSE90761 microarray data and TCGA HNC radiotherapy data were collected for gene set variation analysis (GSVA). “C5.GO.BP.v7.2. Symbols” was used as the reference gene set. The pathways with FDR <0.25 and *p* < 0.05 were considered significantly enriched.

### Cell counting kit-8 assay

Following the manufacturer's instructions, cell viability was examined using cell counting kit-8 (CCK-8; Solarbio, Beijing, China). In brief, TU686 cells overexpressing COL4A1- or empty vector or treated with negative control siRNA or siCOL4A1 were seeded in a 96-well plate (3000 cells/well) for 0, 12, 24, or 48 h. At each time point, 10 μL of CCK-8 solution was added to each well and incubated for another 1 h at 37°C. The absorbance at 450 nm was determined using a microplate reader (FilterMax F3).

### Transwell migration and invasion assay

TU686 cells (300 μL) overexpressing COL4A1- or empty vector or treated with negative control siRNA or siCOL4A1 were seeded in Matrigel (BD, San Jose, CA)-coated upper chamber of a 24-well Transwell device (Corning, NY) at a density of 1.5 × 10^4^ cells/well. The lower chamber was filled with 600 μL Dulbecco's modified Eagle's medium containing 10% FBS. After incubation at 37°C for 24 h, cells in the upper chamber were removed with a sterile cotton swab, whereas cells in the lower surface were fixed in 4% paraformaldehyde for 30 min and stained with 0.1% crystal violet. The stained cells were counted in five randomly selected fields at 10 × magnification. Images were acquired under an inverted light microscope (Ts2-FL; Nikon).

The same setup as described for the invasion assay was used for the migration assay except for the Matrigel coating part.

### Flow cytometry

Early and late cell apoptosis was analyzed using flow cytometry. TU686 cells were collected 48 h after transfection and centrifuged at 800 rpm for 5 min. After washing with ice-cold phosphate-buffered saline, 100,000 cells were reconstituted in 195 μL binding buffer (Beyotime, Shanghai, China) and stained with the mixture of 5 μL of Annexin V-FITC (Beyotime) and 10 μL of propidium iodide for 15 min in the dark. Flow cytometry analysis was performed on a CytoFLEX flow cytometer (Beckman Coulter, Pasadena, CA).

### Quantitative reverse transcription–polymerase chain reaction

Total RNA was isolated using TriQuick reagent (Solarbio). RNA concentration was determined using an ultra-micro nucleic acid detector. Reverse transcription was performed using a MonScript™ RTIII all-in-one mix (Monad Biotech, Hubei, China), followed by polymerase chain reaction (PCR) amplification using a universal SYBR qPCR master mix (Biosharp, Anhui, China) on an AriaMx Real-Time PCR system (Agilent, Santa Clara, CA). β-Actin was used as an internal reference. The PCR primer sequences (5′→3′) were as follows: β-actin, GTCATTCCAAATATGAGATGCGT (forward) and GCTATCACCTCCCCTGTGTG (reverse); COL4A1, AGAAATAGGTTTCCCAGGGCAG (forward) and ATGGATTTGAAAAAGCAATGGCA (reverse). Each reaction was performed in triplicate. The relative gene expression was calculated using the 2^−ΔΔCt^ method.

### Western blot

TU686 cells were collected 72 h after transfection and lysed with RIPA buffer (Solarbio). Protein concentrations were measured using a BCA protein assay kit (Solarbio). Proteins (40 μg) were separated on 10% sodium dodecyl sulfate (SDS) gel and transferred to a polyvinylidene fluoride membrane, followed by 1 h of blocking with 5% skim milk. The membrane was then incubated with anti-COL4A1 antibody (1:1000; Affinity Biosciences, Cincinnati, OH) and β-actin antibody (1:40,000; Abcam) overnight at 4°C, followed by three washes with Tris-buffered saline containing 0.1% Tween-20 (TBST). The membrane was then incubated with horseradish peroxidase–conjugated secondary antibody (1:10,000) for 1 h at room temperature. After three additional washes with TBST, the protein bands were visualized using an enhanced chemiluminescence reagent (Biosharp) and analyzed using ImageJ (NIH, Bethesda, MD).

### Statistical analysis

Data were expressed as mean and standard deviation. Statistical analysis was performed using R software version 3.4.0.3. The topTable and decideTest functions of the limma package were used to summarize the linear model results, perform hypothesis tests, and adjust *p-*values for multiple tests. A value of *p* < 0.05 was considered statistically significant.

## Results

### Identification of radiosensitivity-related DEGs in HNC

To identify radiosensitivity-related DEGs in HNC, we analyzed mRNA expression profiles of the GSE9714 dataset containing 4 radioresistant and 4 radiosensitive cases, the GSE90761 dataset containing 6 radioresistant and 3 radiosensitive cases, and the TCGA dataset containing 292 radiation-treated and 214 nonradiation-treated cases. We found 2248 upregulated and 579 downregulated DEGs in the GSE9714 dataset (Supplementary Fig. S1A, B), 3622 upregulated and 649 downregulated DEGs in the GSE90761 dataset (Supplementary Fig. S1C, D), and 1435 upregulated and 780 downregulated DEGs in the TCGA dataset (Supplementary Fig. S1E, F).

### Identification and characterization of ferroptosis-related DEGs

**FIG. 1.**
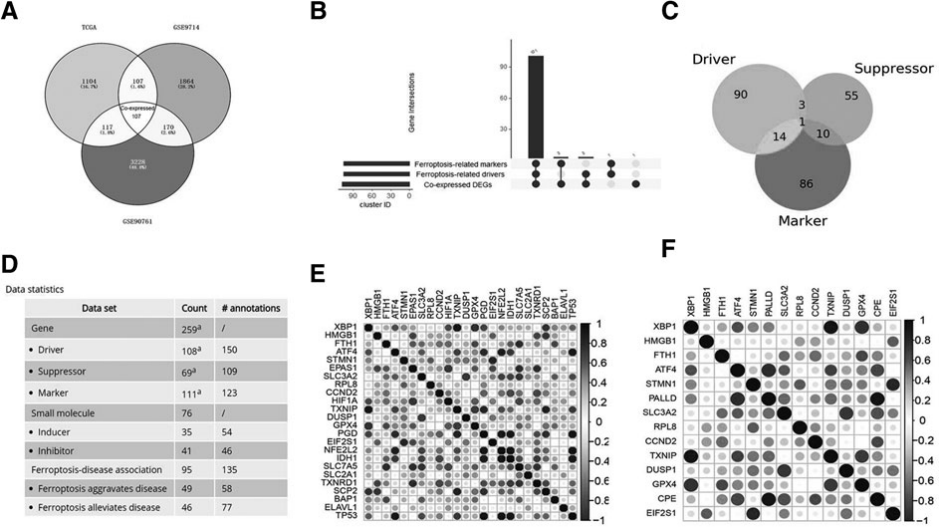
Identification of ferroptosis-related DEGs. **(A)** A Venn diagram depicts the common DEGs among the three datasets. **(B)** The intersection of the ferroptosis markers, ferroptosis driver genes, and the common DEGs. **(C)** A Venn diagram shows the intersection of ferroptosis drivers, ferroptosis suppressors, and ferroptosis markers. **(D)** Statistics of the ferroptosis-related genes. **(E)** Correlation analysis of common DEGs and ferroptosis-related genes. **(F)** Correlation analysis of common DEGs and ferroptosis driver genes. *Blue* represents positive correlations. *Red* represents negative correlations. DEGs, differentially expressed genes.

We further sought to identify ferroptosis-related DEGs. A Venn diagram shows 107 common DEGs among the three datasets (Fig. 1A). The intersection of ferroptosis markers, ferroptosis driver genes, and common DEGs is given in Figure 1B. A Venn diagram shows the intersection of 111 ferroptosis markers, 108 ferroptosis drivers, and 69 ferroptosis suppressors (Fig. 1C, D).

Next, the correlations between common DEGs and ferroptosis markers as well as common DEGs and ferroptosis drivers were analyzed. *PALLD, CCND2, FBLN5, TCF4, ALDOB,* and *NCF1C* were significantly and negatively correlated with ferroptosis markers, with *R* values of −0.860756583, −0.81298762, −0.883872258, −0.761827078, −0.714366877, and −0.8022271, respectively, whereas NLGN1, *CELA2A, NID2,* and *ENO2* were significantly and positively correlated with ferroptosis markers, with *R* values of 0.981497452, 0.788707686, 0.75220168, and 0.741032963, respectively (Supplementary Table S1 and Fig. 1E). *IGFBP4, TIMP1, CD14,* and *LUM* were significantly and positively correlated with ferroptosis drivers (all *R* > 0.8 and *p* < 0.05) (Supplementary Table S2 and Fig. 1F). Common DEGs that were significantly correlated with both ferroptosis markers and drivers are given in Supplementary Table S3.

**FIG. 2.**
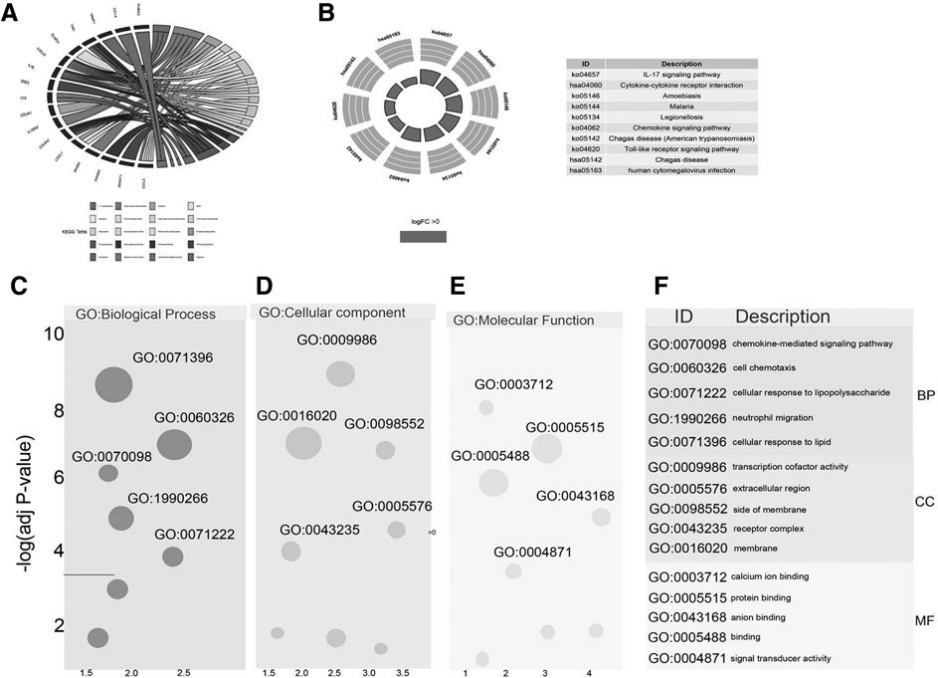
Functional annotation of ferroptosis-correlated DEGs. **(A, B)** KEGG analysis. **(C–F)** GO pathway enrichment analysis. GO, gene ontology; KEGG, Kyoto Encyclopedia of Genes and Genomes.

KEGG pathway analysis revealed that these DEGs are mainly enriched in IL-17, cytokine-cytokine receptor interaction, Toll-like receptor, tumor necrosis factor, focal adhesion signaling pathways, and other classic carcinogenic pathways (Supplementary Table S4 and Fig. 2A, B). GO enrichment analysis showed that the DEGs are mainly enriched in the chemokine-mediated signaling pathway, leukocyte chemotaxis, response to chemokine, cellular response to chemokine cytokine chemotaxis, cellular response to lipid, cellular response to lipopolysaccharide fatty acid metabolism, and other biological pathways (Supplementary Table S5 and Fig. 2C–F).

Then, we conducted GSEA between radiation-treated and nonradiation-treated patients in the TCGA dataset. The enriched pathways are summarized in Supplementary Table S6, including those positively correlated with radiosensitivity (erythematosus, tight junction, intestinal immune network for IgA production, and spliceosome; Supplementary Fig. S2A–D) and those negatively correlated with radiosensitivity (actin cytoskeleton, chemokine signaling pathway, gap junction, and extracellular matrix-receptor interaction [ECM]; Supplementary Fig. S2E–H).

### Identification of hub genes

To explore the association between ferroptosis-related DEGs and clinical traits, we performed WGCNA on the TCGA dataset. We identified four gene modules that were assigned to different colors (Supplementary Fig. S3A). The cluster dendrogram is shown in Supplementary Figure S3B; the associations with the clinical traits were assigned to two colors. TCGA-D6-A6ES sample was excluded from the analysis owing to poor quality (Supplementary Fig. S3C). The correlation between gene modules and clinical traits is given in Supplementary Figures S3D, E. The correlation coefficients were determined (Supplementary Fig. S3F). The brown module containing eight genes (*EMP3, ISG20, IFI27, CD14, BST2, IFI44L, IFI6,* and *IGFBP6*) showed the strongest correlation with the radiation sensitivity of HNC.

Next, we constructed a PPI network to identify the hub genes among the 107 ferroptosis-related DEGs shared by three datasets (Supplementary Fig. S4A). The network of core molecules was established (Supplementary Fig. S4B, C), the interaction between the genes was sorted, and the top 25 hub genes were identified (Supplementary Fig. S4D). Next, we compared the expression of the hub genes between HNC and normal adjacent tissues in the TCGA dataset. The mRNA levels of *COL6A2, LUM, TERM1, TNFAIP6, IL-36G, IFI6, CCL2, SERPINE2, THBS2, MMP10, IL-1B, CXCL8, INHBA, COL4A1,* and *BST2* were significantly higher in HNC samples than in normal adjacent tissue samples (Supplementary Fig. S5A–O).

### Identification of diagnostic and prognostic predictors

**FIG. 3.**
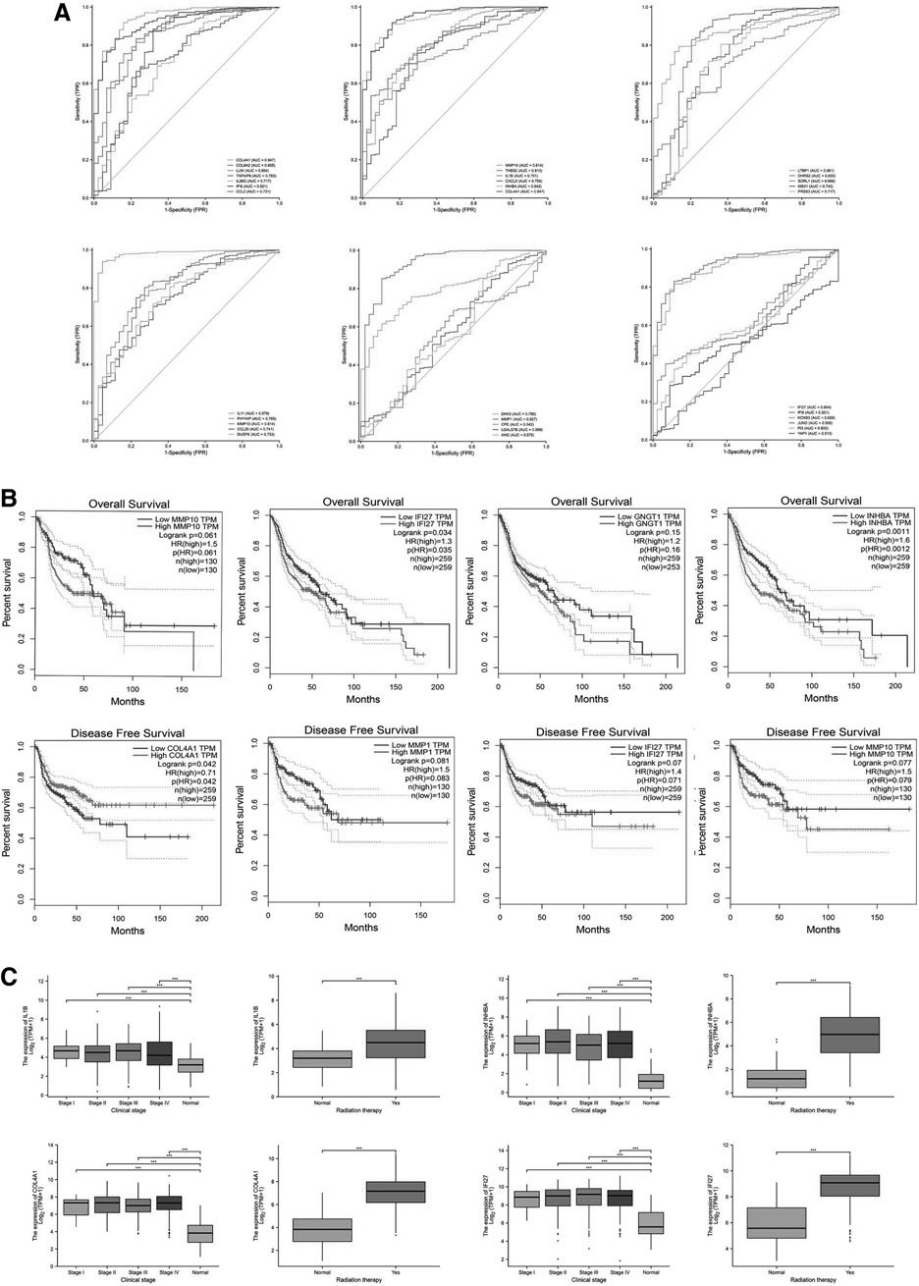
ROC curves. **(A)** ROC curves were generated to assess the diagnostic potentials of hub genes in the TCGA dataset. Kaplan–Meier survival analysis. Patients in the TCGA dataset were divided into high-expression and low-expression groups based on the difference in hub gene expression between cancer tissues and normal adjacent tissues. **(B)** Kaplan–Meier survival curves were generated to assess the correlation of *MMP10, IFI27, GNGT1,* and *INHBA* with the OS of patients, as well as the correlation of *COL4A1 and MMP1* with DFS of patients. **(C)** Comparison of hub gene expression between patients with different cancer stages and radiotherapy status. mRNA levels of *IL-1B*, *INHBA*, *COL4A1*, and *IFI27* were compared between HNC patients in the TCGA dataset with different clinical stages and radiotherapy statuses. Data were expressed as mean ± SD. ***p < 0.001 versus normal. COL4A1, collagen type IV, alpha1 chain; DFS, disease-free survival; HNC, head and neck cancer; OS, overall survival; ROC, receiver operating characteristic; SD, standard deviation; TCGA, The Cancer Genome Atlas.

To assess the diagnostic and prognostic potential of the hub genes, we performed ROC curve analysis and Kaplan–Meier survival analysis. The results of ROC curve analysis (Fig. 3A) showed that *COL6A1, LUM, TNFAIP6, IL-36G, IFI6, CCL2, THBS2, MMP10, IL-1B, CXCL8, INHBA, COL4A1, LTBP1, DHRS2, SORL1, COL4A1, MSX1, PRSS3, IL-11, PHYHIP, MMP10, CCL20, LUM, DUSP6, DKK3, MMP1, CPE, LGALSTB, ANG, IFI27, IFI6, KCNS3, JUND, YAP1,* and *PI3* are potential diagnostic predictors (all area under curve [AUC] >0.50, all *p* < 0.05). Based on the AUC values, *MMP10* (AUC = 0.814), *MMP1* (AUC = 0.927), COL4A1 (AUC = 0.947), *IFI27* (AUC = 0.904), *INHBA* (AUC = 0.942), and *IFI6* (AUC = 0.921) had the greatest diagnostic potential and were subjected to Kaplan–Meier survival analysis to determine their prognostic values.

We then classified patients in the TCGA dataset into high-expression and low-expression groups based on the difference in hub gene expression between cancer tissues and normal adjacent tissues. We found that high *MMP10* (hazards ratio [HR] = 1.5), *IFI27* (HR = 1.3), *GNGT1* (HR = 1.2), *INHBA* (HR = 1.6), and *MMP1* (HR = 1.5) levels were significantly correlated with shorter OS or DFS, suggesting that these five genes are adverse prognostic predictors of HNC (Fig. 3B). Furthermore, *IL-1B, IFI27, INHBA,* and *COL4A1* mRNA levels were significantly increased in TCGA patients with advanced clinical stages or receiving radiotherapy (Fig. 3C and Supplementary Table S7). These data suggest that these hub genes are associated with advanced stages and poor prognosis in HNC.

### Argonaute 1 and ATM transcription factors are correlated with the hub genes

**FIG. 4.**
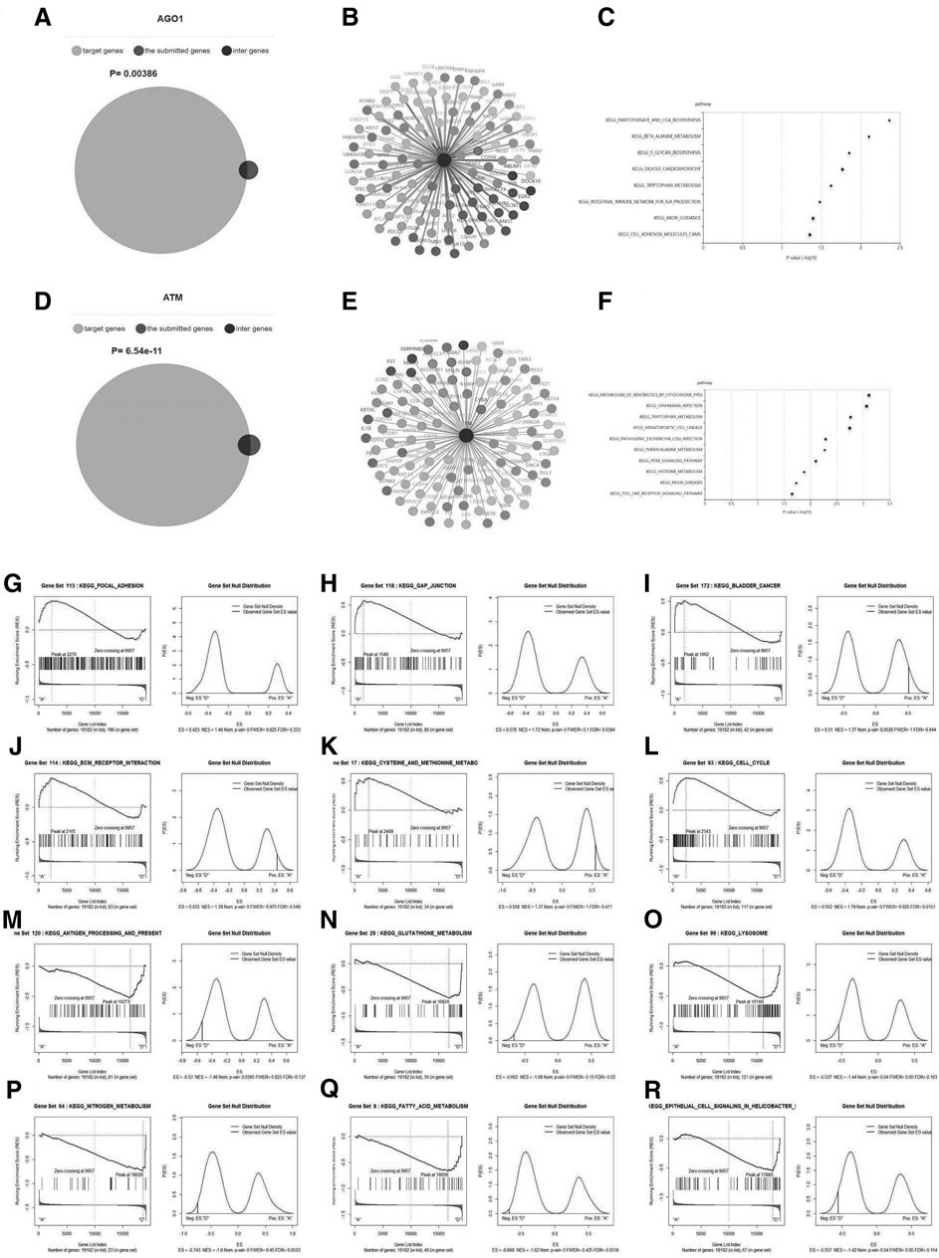
Identification of transcription factors correlated with ferroptosis-correlated DEGs. **(A)** Venn diagram shows the intersecting between AGO1 target genes and ferroptosis-correlated DEGs. **(B, C)** Interaction network and KEGG pathway enrichment analysis of AGO1 target genes. **(D)** A Venn diagram shows the intersecting between ATM target genes and ferroptosis-correlated DEGs. **(E, F)** Interaction network and KEGG pathway enrichment analysis of ATM target genes. Gene set enrichment analysis (GSEA) of AGO1 and ATM. AGO1 was positively correlated with focal adhesion **(G)**, gap junction **(H)**, and bladder cancer **(I)** and negatively correlated with antigen processing and presentation **(M)**, glutathione metabolism **(N)**, and lysosome **(O)**. ATM was positively correlated with ECM receptor interaction **(J)**, cysteine and methionine metabolism **(K)**, and cell cycle **(L)** and negatively correlated with nitrogen metabolism **(P)**, fatty acid metabolism **(Q)**, and cell signaling **(R)** in *Helicobacter pylori*. AGO1, Argonaute 1; ATM, Acute Transverse Myelitis; ECM, extracellular matrix-receptor interaction.

To explore the potential regulators of the hub genes, we sought to identify the transcription factors correlated with the hub genes. The results showed that AGO1 (Fig. 4A, *p* = 0.00386) and ATM (Fig. 4D, *p* = 6.54e-11) significantly correlated with the hub genes. The PPI network of Argonaute 1 (AGO1) (Fig. 4B) and Acute Transverse Myelitis (ATM) (Fig. 4E) were constructed. KEGG analysis showed that AGO1 and ATM were mainly involved in focal adhesion, gap junction invasion, and metastasis pathways in HNC (Fig. 4C and 4F). The results of GSEA revealed that AGO1 was positively correlated with focal adhesion (Fig. 4G), gap junction (Fig. 4H), and bladder cancer (Fig. 4I), but negatively correlated with antigen processing and presentation (Fig. 4M), glutathione metabolism (Fig. 4N), and lysosome (Fig. 4O). ATM was positively correlated with ECM receptor interaction (Fig. 4J), cysteine and methionine metabolism (Fig. 4K) and cell cycle (Fig. 4L) but negatively correlated with nitrogen metabolism (Fig. 4P), fatty acid metabolism (Fig. 4Q), and cell signaling by *Helicobacter pylori* (Fig. 4R).

### Gene set variation analysis

**FIG. 5.**
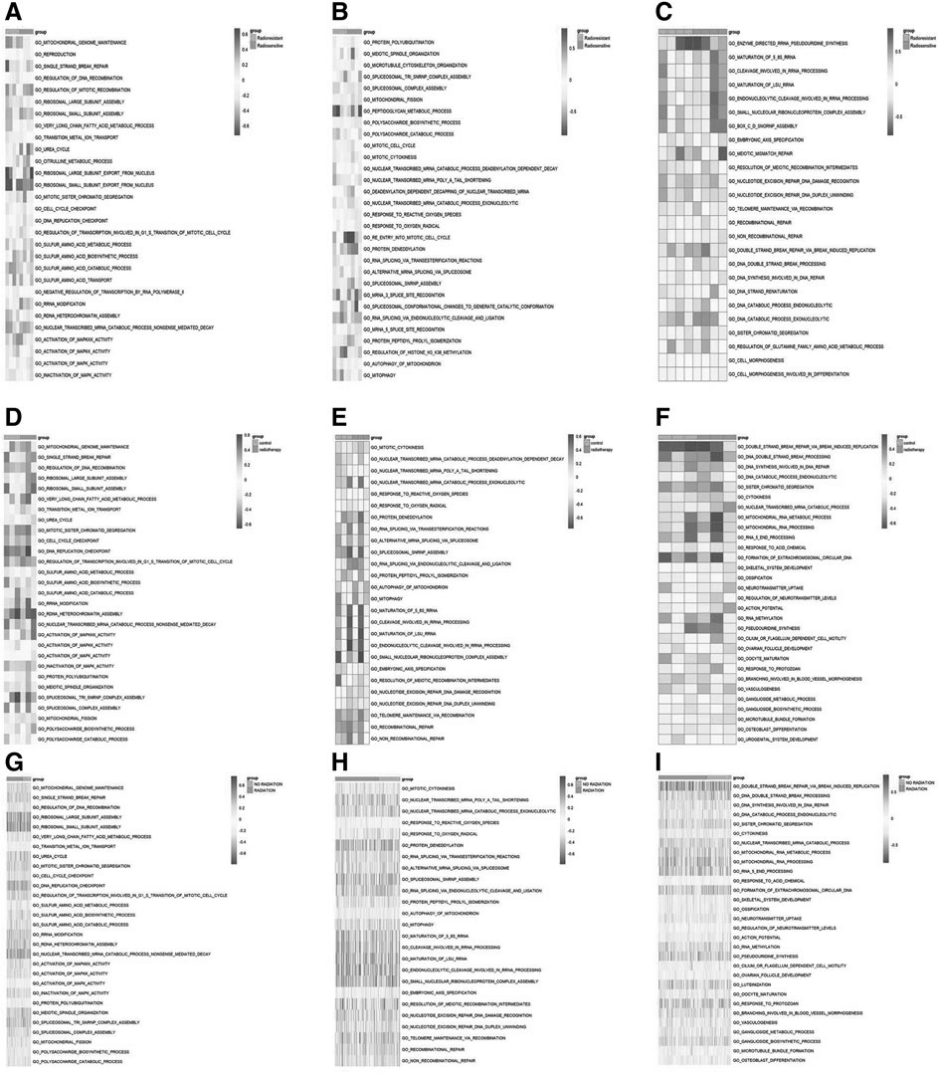
GSVA. GSVA of the GSE9714 **(A–C)**, GSE97061 **(D–F)**, and TCGA **(G–I)** datasets. *Red* indicates high expression. *Blue* indicates low expression. GSVA, gene set variation analysis.

We performed GSVA using a single sample from the GSE9714, GSE90761, and TCGA datasets. The results showed that the most involved pathways in the GSE9714 dataset were transition metal ion transport and ribosomal large subunit export from the nucleus, ribosomal small subunit export from the nucleus, nucleus transports and mitotic sister chromatid segregation, cell cycle checkpoint, DNA replication checkpoint, regulation of nucleus transport and transcription factors involved in the G1 to S phase transition during the mitotic cell cycle, sulfur amino acid biosynthetic carcinogenesis, cell cycle, DNA damage repair, and other biological pathways (Fig. 5A–C). The most involved pathways in the GSE90761 dataset were regulation of DNA recombination, ribosomal large subunit assembly, ribosomal small subunit assembly, nucleic acid assembly, nuclear material renewal, and transport, very long chain fatty acid and iron ion transport, oxidative stress, fatty acid metabolism, and other biological processes were the most significant pathways (Fig. 5D–F).

The most involved pathways in the TCGA dataset were the G1 to S phase transition during the mitotic cycle, amino acid metabolism, sulfur-containing amino acid biosynthesis, RNA modification, and other biological processes such as cell cycle, RNA modification, and amino acid metabolism (Fig. 5G–I).

### *COL4A1, MMP10,* and *INHBA* expression are negatively correlated with immune infiltration in HNC

**FIG. 6.**
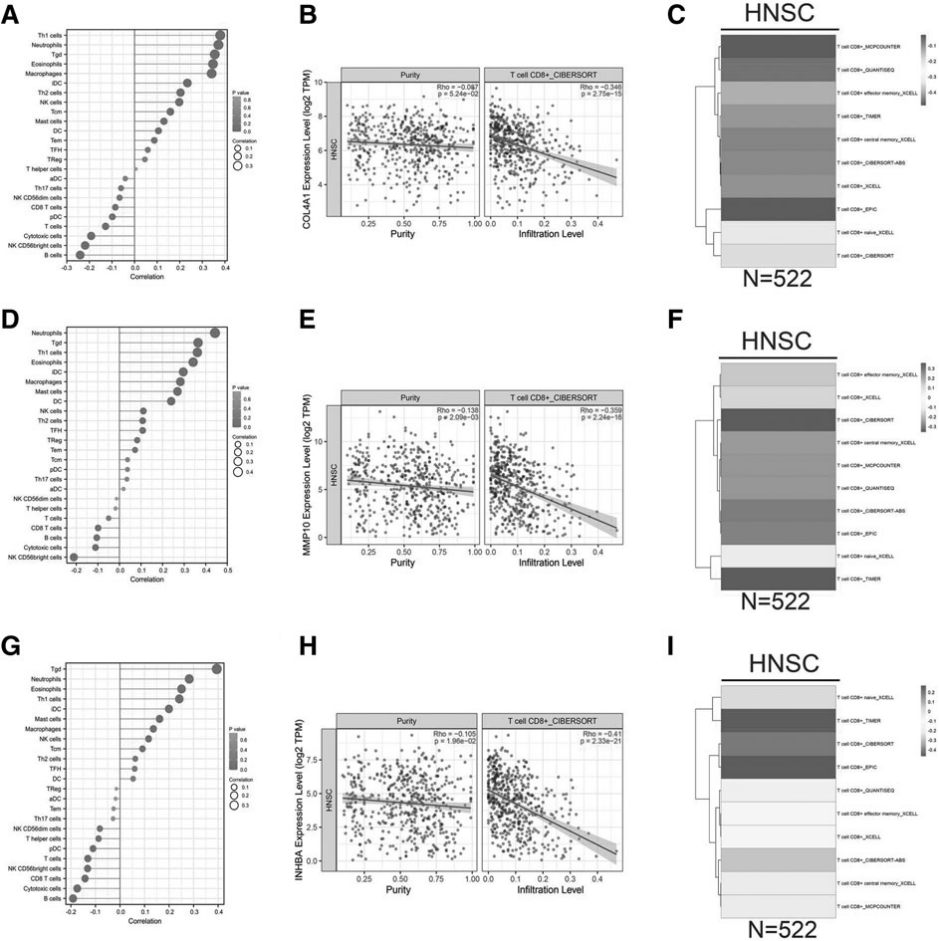
Correlation of hub genes with immune infiltration in HNC. **(A, D, G)** Correlation of *COL4A1* **(A)**, *MMP10* **(D)**, and *INHBA* **(G)** with different immune cells in the TCGA dataset. **(B, E, H)** Correlation of *COL4A1* **(B)**, *MMP10* **(E)**, and *INHBA* **(H)** with CD8^+^ T cells in the TCGA dataset. **(C, F, I)** Correlations between COL4A1/MMP10/INHBA expression and immune infiltration were examined using CIBERSORT, EPIC, TIMER, and XELL tools on the TIMER2.0 website.

To investigate the role of the prognostic hub genes (*INHBA, MMP10, COL4A1, IFI27,* and *MMP1*) in immune infiltration, we assessed their correlations with various immune cells in the TCGA database (Fig. 6A, D, G). Our results showed that expression of *COL4A1, MMP10,* and *INHBA* were negatively correlated with CD8^+^ T cell proportion, with correlation coefficients of −0.346, −0.359, and −0.41, respectively (Fig. 6B, E, H). Figure 6C, F, and I show their correlations with other immune cells. These data suggest that HNC tumors with higher *COL4A1, MMP10,* and *INHBA* expression may have a lower immune response and are more likely to invade and metastasize.

### Knockdown of *COL4A1* inhibits cell proliferation, migration, and invasion while promoting apoptosis in human laryngeal cancer cells

**FIG. 7.**
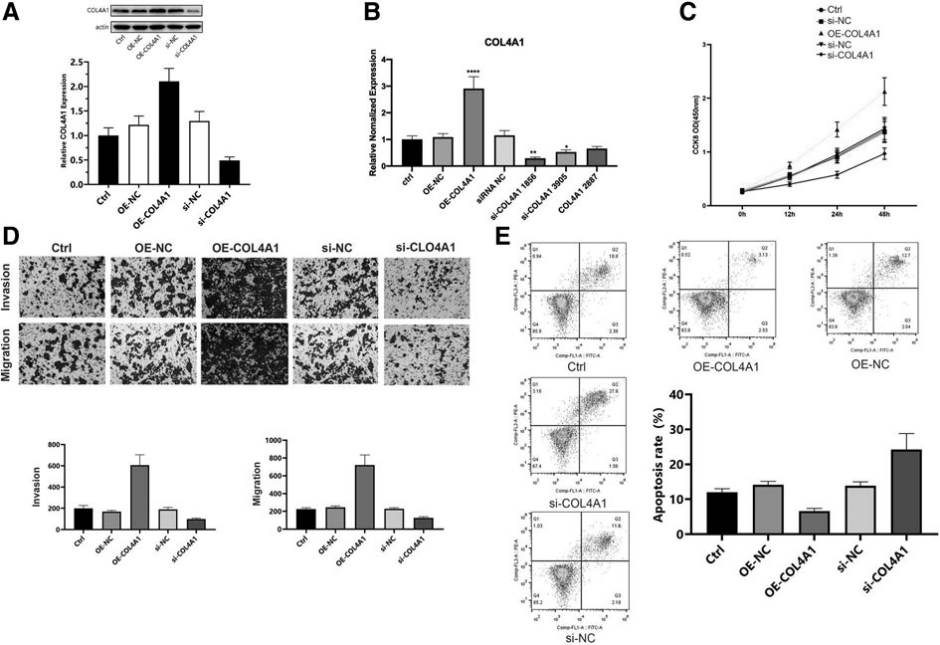
Knockdown of COL4A1 inhibited cell proliferation, migration, and invasion while promoting apoptosis in TU686 cells. **(A, B)** TU686 human laryngeal cancer cells were transfected with vectors overexpressing COL4A1 or siRNA against COL4A1. Western blot and qRT-PCR were performed to measure COL4A1 protein and mRNA levels. Data were expressed as mean ± SD. *p < 0.05, **p < 0.01, ***p < 0.001 versus NC. **(C)** CCK-8 assay was conducted to examine cell proliferation. **(D)** A Transwell assay was carried out to examine cell migration and invasion. **(E)** Flow cytometry analysis was performed to determine cell apoptosis. CCK-8, cell counting kit-8; NC, negative control; qRT-PCR, quantitative reverse transcription–polymerase chain reaction; si, small interfering RNA.

To validate whether hub genes contribute to HNC development, we selected one of the genes, that is, *COL4A1*, and examined its effects on cell proliferation, migration, invasion, and apoptosis in human laryngeal cancer cells. As given in Figure 7A and B, when COL4A1 was overexpressed, the mRNA and protein levels of COL4A1 in TU686 cells increased; after silencing, the mRNA and protein levels decreased. Knockdown of COL4A1 significantly inhibited cell proliferation, migration, and invasion (Fig. 7C, D) while promoting apoptosis (Fig. 7E), whereas overexpression of COL4A1 exhibited opposite effects. These results suggest that hub genes identified in this study may serve as therapeutic targets for HNC.

## Discussion

In this study, we analyzed the GEO and TCGA HNC databases and identified 107 common DEGs associated with radiosensitivity. The WGCNA showed that *EMP3, ISG20, IFI27, CD14, BST2, IFI44L, IFI6,* and *IGFBP6* had the strongest correlation with radiosensitivity in patients with HNC. KEGG pathway analysis further showed that these DEGs were significantly enriched in IL-17, PI3K-Act, and NF-κB signaling pathways. GO enrichment analysis demonstrated a close relationship between DEGs and chemokines. Among the top 25 hub genes identified by the PPI network, *MMP10, MMP1, COL4A1, IFI27*, and *INHBA* showed the highest diagnostic and prognostic value, and *COL4A1, MMP10*, and *INHBA* expressions were negatively correlated with immune infiltration, serving as promising therapeutic targets for HNC.

Radiation therapy is the main treatment strategy for Nasopharyngeal carcinoma (NPC) patients, and prognoses are mainly based on clinical stage. In this study, we conducted WGCNA and found that *EMP3, ISG20, IFI27, CD14, BST2, IFI44L, IFI6,* and *IGFBP6* are the most relevant genes associated with radiotherapy in patients with HNC. Zheng et al. (2017) reported that *EMP3* suppression can inhibit the metastasis of oral squamous cancer cells and that its downregulation contributes to the chemoresistance and radioresistance of breast cancers. Chen et al. (2016) have shown that the expression of *IGFBP6* in nasopharyngeal carcinoma is linked to a reduced local recurrence rate and a reduced risk of long-distance metastasis. They suggested that more aggressive treatment, such as radiotherapy or targeted chemotherapy, may be advised in NPC patients negative for IGFBP6, who are more likely to experience distant metastasis (Chen et al., 2016).

Fang et al. (2014) suggested that a high level of *BST2* expression can facilitate the migration of oral cavity cancer. Also, Kuang et al. (2017) discovered that upregulation of BST2 results in platinum resistance in patients with locally advanced NPC by activating the NF-κB pathway, which regulates tumorigenicity, proliferation, chemoresistance, and radioresistance of multiple kinds of cancers, including NPC (Zhan and Fan, 2020). Hsu et al. (2019) reported that the knockout of podoplanin increases the expression of *IFI27, IFI44L,* and *IFI6* and reduces the proliferation, migration, and invasion of nasopharyngeal carcinomas. Guo et al. (2012) combined GO and PPI networks and identified several genes, including IFI27, involved in the radioresistance of nasopharyngeal carcinoma. To sum up, all these genes may be promising therapeutic targets for HNC.

KEGG enrichment analysis showed that ferroptosis-related DEGs are significantly associated with IL-17, PI3K-Akt, and NF-κB signaling pathways. IL-17B signaling can alter the tumor microenvironment (TME) by promoting chemokine and cytokine secretion, thereby facilitating tumor growth (Bastid et al., 2020). Activation of the PI3K/Akt/β-catenin signaling pathway can promote the proliferation and metastasis of HNC (Zhou et al., 2019a). Moreover, the NF-κB signaling pathway contributes to HNC development by promoting the migration and invasion of cancer cells (Qin et al., 2018). Also, it is well known that chemokines have a critical role in developing HNC (Zhao et al., 2022). In our GO analysis, ferroptosis-related DEGs were significantly associated with response to chemokines, cellular response to chemokines, cell chemotaxis, chemokine-mediated signaling pathway, and granulocyte chemotaxis, suggesting that ferroptosis-related DEGs contribute to HNC occurrence and metastasis through chemokines.

Previous studies have shown that ATM and AGO1 promote the progression of different types of cancer (Fawzy et al., 2020; Lang et al., 2018; Luo et al., 2021; Wang et al., 2018b). However, their roles in HNC are poorly understood, suggesting a novel direction for future research. We found that transcription factors AGO1 and ATM are highly associated with ferroptosis-related DEGs. The results of GSEA showed that AGO1 and ATM are positively correlated with invasion and metastasis pathways such as FOCAL_ADHERIN and GAP_JUNCTION, but negatively correlated with antigen presentation, fatty acid metabolism, oxidative stress, and metabolic output.

Based on the PPI analysis, we identified hub genes that could be used for diagnostic and prognostic purposes for HNC, including *MMP10, MMP1, COL4A1, IFI27,* and *INHBA*. In HNC, overexpression of *INHBA* is closely involved in post-transcriptional regulation and protein translation (Wu et al., 2019). Another study found that *MMP-10* promotes the invasion and metastasis of HNC (Deraz et al., 2011). Reis et al. (2020) suggested a 4-gene signature, including *COL4A1* and *MMP1*, from histologically normal surgical margins that may predict local recurrence in patients with oral carcinoma. Wang et al. (2018a) found that *IFI27* promotes cancer cell proliferation and invasion in oral squamous cell carcinoma. Taken together, these hub genes may contribute to invasion and metastasis in HNC, which is consistent with our findings.

The TME has an important role in the development and prognosis of cancer (Arneth, 2019). Radiotherapy can induce various immune responses within the TME (Ozpiskin et al., 2019). A high proportion of CD8^+^ T cells may increase radiosensitivity and improve survival rates in HNC (Fiedler et al., 2020; Walker et al., 2020). Our results showed that *INHBA, MMP10, COL4A1, IFI27,* and *MMP1* expressions are significantly and negatively correlated with CD8+ T cell proportion in patients who did not receive radiation therapy or had radiation resistance. In addition, *INHBA, MMP10, COL4A1, IFI27,* and *MMP1* expressions were significantly increased in patients with advanced HNC. Therefore, these hub genes may affect the development and progression of HNC by modulating immune infiltration.

Since few studies have examined the role of *COL4A1* in HNC, we further explored the effects of *COL4A1* on HNC cell proliferation, migration, invasion, and apoptosis *in vitro*. Knockdown of *COL4A1* inhibited proliferation, migration, and invasion while promoting apoptosis in TU686 cells. This finding suggests that *COL4A1* is a promising therapeutic target for HNC. In the future, we plan to do the same for *INHBA, MMP10, IFI27, and MMP1* and further investigate the antitumor effect of these genes *in vivo*.

This study first reported ferroptosis-related genes, that is, INHBA, MMP10, COL4A1, IFI27, and MMP1, associated with radioresistant HNCs. These genes are negatively correlated with immune infiltration in HNC and have diagnostic and prognostic potentials; thus, they may serve as promising therapeutic targets for HNC. However, in this study, we did not explore the ferroptosis-related mechanisms, which we plan to do in our future study.

## Data Availability Statement

Data are available in a public, open-access repository. Data are available on reasonable request. All data relevant to the study are included in the article. The datasets (GEO data) and (TCGA data) for this study can be found on the following web link: GEO (www.ncbi.nlm.nih.gov/geo/and TCGA (https://portal.gdc.cancer.gov/).

## Ethics Approval Statement

Our study did not require ethical board approval because it did not contain human or animal trials.

## Supplementary Material

Supplemental data

Supplemental data

Supplemental data

Supplemental data

Supplemental data

Supplemental data

Supplemental data

Supplemental data

Supplemental data

Supplemental data

Supplemental data

Supplemental data
