# Supplementary material for: Ferroptosis-Related Genes Are Associated with Radioresistance and Immune Suppression in Head and Neck Cancer
Source: Genet Test Mol Biomarkers. 2024 Mar 28;28(3):100–13. doi: 10.1089/gtmb.2023.0193 (PMC10979683; doi:10.1089/gtmb.2023.0193)

**Figure S1. Identification of DEGs associated with radiosensitivity in head and neck cancer (HNC).** mRNA expression profiles of HNC datasets were downloaded from The Cancer Genome Atlas (TCGA) and Gene Expression Omnibus (GEO). Genes with |log fold change (logFC)| > 1 were considered DEGs between radioresistant and radiosensitive HNC. **(A, B)** Heatmap (A) and volcano map (B) of gene expression of the GSE9714 dataset (4 radioresistant cases and 4 radiosensitive cases). Brown indicates upregulated DEGs. Purple indicates downregulated DEGs. **(C, D)** Heatmap (C) and volcano map (D) of gene expression of the GSE97061 dataset (6 radioresistant cases and 3 radiosensitive cases). Brown indicates upregulated DEGs. Pink indicates downregulated DEGs. **(E, F)** Heatmap (E) and volcano map (F) of gene expression of the TCGA dataset (292 radiation-treated and 214 non-radiation-treated cases). Peach indicates upregulated DEGs. Turquoise indicates downregulated DEGs. Black indicates genes that have not changed significantly.


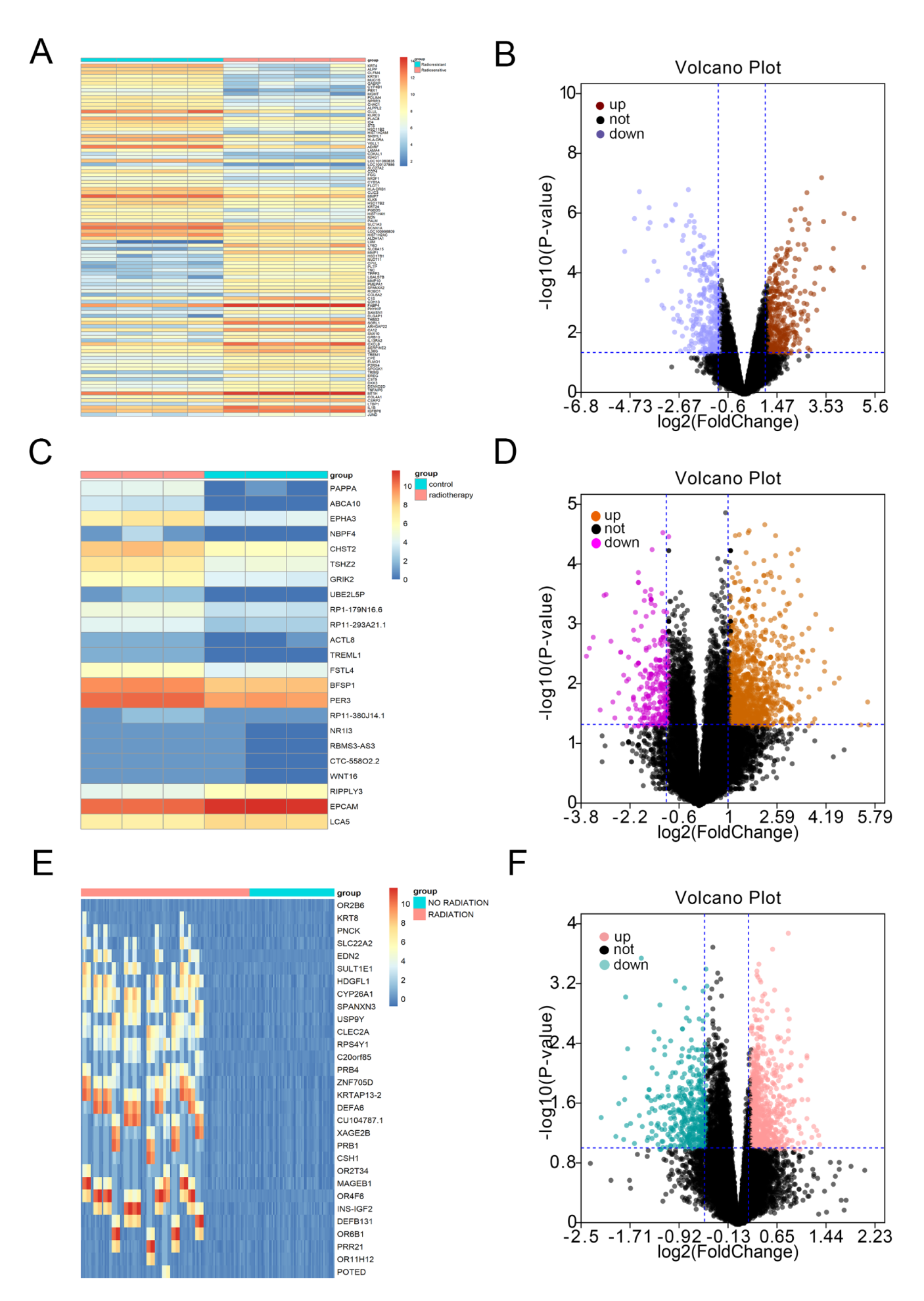

Supplement: Supplemental data [file Suppl_FigureS1.docx]
