# Supplementary material for: Ferroptosis-Related Genes Are Associated with Radioresistance and Immune Suppression in Head and Neck Cancer
Source: Genet Test Mol Biomarkers. 2024 Mar 28;28(3):100–13. doi: 10.1089/gtmb.2023.0193 (PMC10979683; doi:10.1089/gtmb.2023.0193)
Supplement: Supplemental data [file Suppl_TableS1.docx]

**Table S1 Primer sequences of the key miRNAs**

| Gene | Primer sequence (5^’^-3^’^) |
| --- | --- |
| ACTB-F | GTCATTCCAAATATGAGATGCGT |
| ACTB-R | GCTATCACCTCCCCTGTGTG |
| COL4A1-F | AGAAATAGGTTTCCCAGGGCAG |
| COL4A1-R | ATGGATTTGAAAAAGCAATGGCA |

F: forward primer; R: reversed primer
