# Supplementary material for: Ferroptosis-Related Genes Are Associated with Radioresistance and Immune Suppression in Head and Neck Cancer
Source: Genet Test Mol Biomarkers. 2024 Mar 28;28(3):100–13. doi: 10.1089/gtmb.2023.0193 (PMC10979683; doi:10.1089/gtmb.2023.0193)
Supplement: Supplemental data [file Suppl_TableS2.docx]

**Table S2. Ferroptosis-related marker genes**.

| GENE1 | GENE2 | P | R |
| --- | --- | --- | --- |
| PALLD | PGD | 0.01 | -0.86 |
| CCND2 | IDH1 | 0.01 | -0.81 |
| ENO2 | RPL8 | 0.04 | 0.74 |
| FBLN5 | KEAP1 | 0.00 | -0.88 |
| TCF4 | EPAS1 | 0.03 | -0.76 |
| NID2 | ELAVL1 | 0.03 | 0.75 |
| ALDOB | PHKG2 | 0.05 | -0.71 |
| WIF1 | MAPK9 | 0.04 | -0.74 |
| NCF1C | TP53 | 0.02 | -0.80 |
| NLGN1 | CDO1 | 1.56E-05 | 0.98 |
| CELA2A | HIF1A | 0.02 | 0.79 |
| COA1 | ALOX5 | 0.05 | -0.71 |
| IL24 | EGFR | 0.01 | 0.84 |
| APOF | WIPI1 | 0.04 | -0.73 |
| HTR2C | CHAC1 | 0.02 | -0.81 |
| GLCE | ACSL4 | 0.01 | 0.85 |
| SLC5A12 | HMOX1 | 0.01 | 0.82 |
| MAGEA2B | TNFAIP3 | 0.02 | 0.79 |
| SYNPO2L | LPCAT3 | 0.02 | 0.78 |
| KLF3-AS1 | MAPK8 | 0.01 | -0.85 |
| MAK | MAPK14 | 0.01 | -0.81 |
