# Supplementary material for: Ferroptosis-Related Genes Are Associated with Radioresistance and Immune Suppression in Head and Neck Cancer
Source: Genet Test Mol Biomarkers. 2024 Mar 28;28(3):100–13. doi: 10.1089/gtmb.2023.0193 (PMC10979683; doi:10.1089/gtmb.2023.0193)
Supplement: Supplemental data [file Suppl_TableS3.docx]

**Table S3. Ferroptosis-related driver genes**.

| GENE1 | GENE2 | P | R |
| --- | --- | --- | --- |
| CPE | HMOX1 | 0.00 | 0.88 |
| CPE | AURKA | 0.02 | 0.78 |
| CPE | HSD17B11 | 0.02 | 0.78 |
| IGFBP4 | HSPB1 | 0.00 | 0.89 |
| IGFBP4 | CAPG | 0.01 | 0.82 |
| IGFBP4 | SLC2A6 | 0.00 | 0.93 |
| TIMP1 | HSD17B11 | 0.05 | 0.71 |
| TIMP1 | SLC2A6 | 0.01 | 0.85 |
| CD14 | ALOX12 | 0.05 | 0.71 |
| CD14 | SLC2A6 | 0.03 | 0.76 |
| LUM | HSPB1 | 0.05 | 0.71 |
| LUM | HMOX1 | 0.00 | 0.88 |
| LUM | AURKA | 0.01 | 0.86 |
| LUM | CXCL2 | 0.02 | 0.80 |
| LUM | NCF2 | 0.02 | 0.78 |
| LUM | HSD17B11 | 0.02 | 0.79 |
| LUM | SLC2A6 | 0.03 | 0.75 |
| BASP1 | NCF2 | 0.00 | 0.88 |
| BASP1 | HSD17B11 | 0.02 | 0.78 |
| BASP1 | SLC2A6 | 0.01 | 0.81 |
| IFI27 | HMOX1 | 0.05 | 0.72 |
| IFI27 | SLC2A6 | 0.04 | 0.72 |
| LTBP1 | HMOX1 | 0.00 | 0.93 |
| LTBP1 | HSD17B11 | 0.03 | 0.74 |
| LTBP1 | SLC2A6 | 0.04 | 0.74 |
| CXCL8 | HSPB1 | 0.01 | 0.84 |
| CXCL8 | RRM2 | 0.02 | 0.81 |
| CXCL8 | HMOX1 | 0.01 | 0.85 |
| CXCL8 | AURKA | 0.00 | 0.94 |
