# Supplementary material for: Ferroptosis-Related Genes Are Associated with Radioresistance and Immune Suppression in Head and Neck Cancer
Source: Genet Test Mol Biomarkers. 2024 Mar 28;28(3):100–13. doi: 10.1089/gtmb.2023.0193 (PMC10979683; doi:10.1089/gtmb.2023.0193)
Supplement: Supplemental data [file Suppl_TableS4.docx]

**Table S4. Differentially expressed genes**.

| DEGs |  | genes |
| --- | --- | --- |
| Ferroptosis driver&marker&co-expressed |  | PALLD CCND2 CPE ENO2 IGFBP4 KDM5B BST2 TNC TIMP1 CD14 LUM PLTP DKK3 SPOCK1 BASP1 IFI27 LTBP1 CXCL8 THBS2 FBLN5 TRAPPC12 S100A4 UPP1 SORL1 PI3 EMP3 JUND TCF4 IGFBP6 CA12 FABP4 P2RX4 NID2 COL16A1 IFI6 IFI44L CXCL1 MMP1 ELMO1 HSD3B1 MMP12 ISG20 ALDOB WIF1 CDH13 PDPN ARHGAP25 INHBA FST SRPX NCF1C IL1B CCL3L3 SGCB ANG AREG CDC14A RBL1 PHYHIP |
