# Supplementary material for: Ferroptosis-Related Genes Are Associated with Radioresistance and Immune Suppression in Head and Neck Cancer
Source: Genet Test Mol Biomarkers. 2024 Mar 28;28(3):100–13. doi: 10.1089/gtmb.2023.0193 (PMC10979683; doi:10.1089/gtmb.2023.0193)
Supplement: Supplemental data [file Suppl_TableS5.docx]

**Table S5. GO pathway analysis for the DEGs**.

| ID | term | Category | adj_pvalue | genes |
| --- | --- | --- | --- | --- |
| GO:0071621 | granulocyte chemotaxis | GO Biological Processes | 3.53E-07 | C3AR1,CXCL1,IL1B,CXCL8,CCL2,TRPV4,CCL3L3 |
| GO:0008285 | negative regulation of cell proliferation | GO Biological Processes | 7.27E-07 | CDH13,EREG,CXCL1,IL1B,CXCL8,INHBA,MSX1,SERPINE2,ROBO1, CCL2,TENM1,PDPN,CCL3L3,LGALS7B |
| GO:0097530 | granulocyte migration | GO Biological Processes | 1.14E-06 | C3AR1,CXCL1,IL1B,CXCL8,CCL2,TRPV4,CCL3L3 |
| GO:0097529 | myeloid leukocyte migration | GO Biological Processes | 1.36E-06 | C3AR1,CXCL1,IL1B,CXCL8,P2RX4,CCL2,TRPV4,CCL3L3 |
| GO:0030593 | neutrophil chemotaxis | GO Biological Processes | 1.82E-06 | C3AR1,CXCL1,IL1B,CXCL8,CCL2,CCL3L3 |
| GO:1990266 | neutrophil migration | GO Biological Processes | 5.11E-06 | C3AR1,CXCL1,IL1B,CXCL8,CCL2,CCL3L3 |
| GO:0050900 | leukocyte migration | GO Biological Processes | 1.54E-05 | C3AR1,CXCL1,IL1B,CXCL8,MMP1,P2RX4,CCL2,TREM1,TRPV4,CCL3L3 |
| GO:0060326 | cell chemotaxis | GO Biological Processes | 1.55E-05 | C3AR1,CXCL1,IL1B,CXCL8,P2RX4,CCL2,TRPV4,CCL3L3 |
| GO:0070098 | chemokine-mediated signaling pathway | GO Biological Processes | 1.55E-05 | CXCL1,CXCL8,ROBO1,CCL2,CCL3L3 |
| GO:0030595 | leukocyte chemotaxis | GO Biological Processes | 1.97E-05 | C3AR1,CXCL1,IL1B,CXCL8,CCL2,TRPV4,CCL3L3 |
| GO:1990868 | response to chemokine | GO Biological Processes | 2.49E-05 | CXCL1,CXCL8,ROBO1,CCL2,CCL3L3 |
| GO:1990869 | cellular response to chemokine | GO Biological Processes | 2.49E-05 | CXCL1,CXCL8,ROBO1,CCL2,CCL3L3 |
| GO:0071396 | cellular response to lipid | GO Biological Processes | 4.65E-05 | CD14,CYP24A1,CXCL1,TNC,IL1B,CXCL8,INHBA,CCL2,IL36G,PMEPA1 |
| GO:0071222 | cellular response to lipopolysaccharide | GO Biological Processes | 9.88E-05 | CD14,CXCL1,IL1B,CXCL8,CCL2,IL36G |
