# Supplementary material for: Ferroptosis-Related Genes Are Associated with Radioresistance and Immune Suppression in Head and Neck Cancer
Source: Genet Test Mol Biomarkers. 2024 Mar 28;28(3):100–13. doi: 10.1089/gtmb.2023.0193 (PMC10979683; doi:10.1089/gtmb.2023.0193)
Supplement: Supplemental data [file Suppl_TableS6.docx]

**Table S6. KEGG pathway analysis for the DEGs**.

| ID | term | category | adj_pval | genes |
| --- | --- | --- | --- | --- |
| ko04657 | IL-17 signaling pathway | KEGG Pathway | 2.03E-05 | CXCL1,IL1B,CXCL8,MMP1,CCL2 |
| hsa04060 | Cytokine-cytokine receptor interaction | KEGG Pathway | 2.21E-05 | CXCL1,IL1B,CXCL8,IL13RA2,INHBA,CCL2,IL36G,CCL3L3 |
| ko05146 | Amoebiasis | KEGG Pathway | 2.37E-05 | CD14,COL4A1,CXCL1,IL1B,CXCL8 |
| ko05144 | Malaria | KEGG Pathway | 2.76E-05 | IL1B,CXCL8,CCL2,THBS2 |
| ko05134 | Legionellosis | KEGG Pathway | 4.37E-05 | CD14,CXCL1,IL1B,CXCL8 |
| ko04062 | Chemokine signaling pathway | KEGG Pathway | 4.72E-05 | GNGT1,CXCL1,CXCL8,CCL2,ELMO1,CCL3L3 |
| ko05142 | Chagas disease (American trypanosomiasis) | KEGG Pathway | 0.00 | IL1B,CXCL8,CCL2,CCL3L3 |
| ko04620 | Toll-like receptor signaling pathway | KEGG Pathway | 0.00 | CD14,IL1B,CXCL8,CCL3L3 |
| hsa05142 | Chagas disease | KEGG Pathway | 0.00 | IL1B,CXCL8,CCL2,CCL3L3 |
| hsa05163 | human cytomegalovirus infection | KEGG Pathway | 0.00 | GNGT1,IL1B,CXCL8,CCL2,CCL3L3 |
| ko04621 | NOD-like receptor signaling pathway | KEGG Pathway | 0.00 | CXCL1,IL1B,CXCL8,CCL2 |
| ko04064 | NF-kappa B signaling pathway | KEGG Pathway | 0.00 | CD14,IL1B,CXCL8 |
| ko04668 | TNF signaling pathway | KEGG Pathway | 0.01 | CXCL1,IL1B,CCL2 |
| ko04974 | Protein digestion and absorption | KEGG Pathway | 0.00 | COL4A1,COL6A2,PRSS2,ATP1B4 |
| ko04512 | ECM-receptor interaction | KEGG Pathway | 0.00 | COL4A1,COL6A2,TNC,THBS2 |
| hsa04151 | PI3K-Akt signaling pathway | KEGG Pathway | 0.00 | COL4A1,COL6A2,EREG,GNGT1,TNC,THBS2 |
| ko04510 | Focal adhesion | KEGG Pathway | 0.01 | COL4A1,COL6A2,TNC,THBS2 |
| ko05150 | Staphylococcus aureus infection | KEGG Pathway | 0.00 | C1R,C1S,C3AR1 |
| ko04610 | Complement and coagulation cascades | KEGG Pathway | 0.00 | C1R,C1S,C3AR1 |
| ko04145 | Phagosome | KEGG Pathway | 0.00 | C1R,CD14,THBS2,TUBA1A |
