# Supplementary material for: Ferroptosis-Related Genes Are Associated with Radioresistance and Immune Suppression in Head and Neck Cancer
Source: Genet Test Mol Biomarkers. 2024 Mar 28;28(3):100–13. doi: 10.1089/gtmb.2023.0193 (PMC10979683; doi:10.1089/gtmb.2023.0193)

**Figure S2 GSEA between** **radiation-treated and non-radiation-treated patients in the** **TCGA dataset.** **(A–D)** Enriched pathways that were positively correlated with radiosensitivity: (A) systemic lupus erythematosus, (B) tight junction, (C) intestinal immune network for IgA production, and (D) spliceosome. **(E–H)** Enriched pathways that were negatively correlated with radiosensitivity: (E) regulation of actin cytoskeleton, (F) chemokine signaling pathway, (G) gap junction, and (H) ECM receptor interactions. An upward kurtosis indicates a positive correlation. Different colors represent different pathways. As shown in the lower left corner, the P and false discovery rate (FDR) values are statistically significant. In the bottom label, the left side represents the radioresistant group, and the right represents the radiosensitive group. *P* < 0.05, FDR < 0.25.


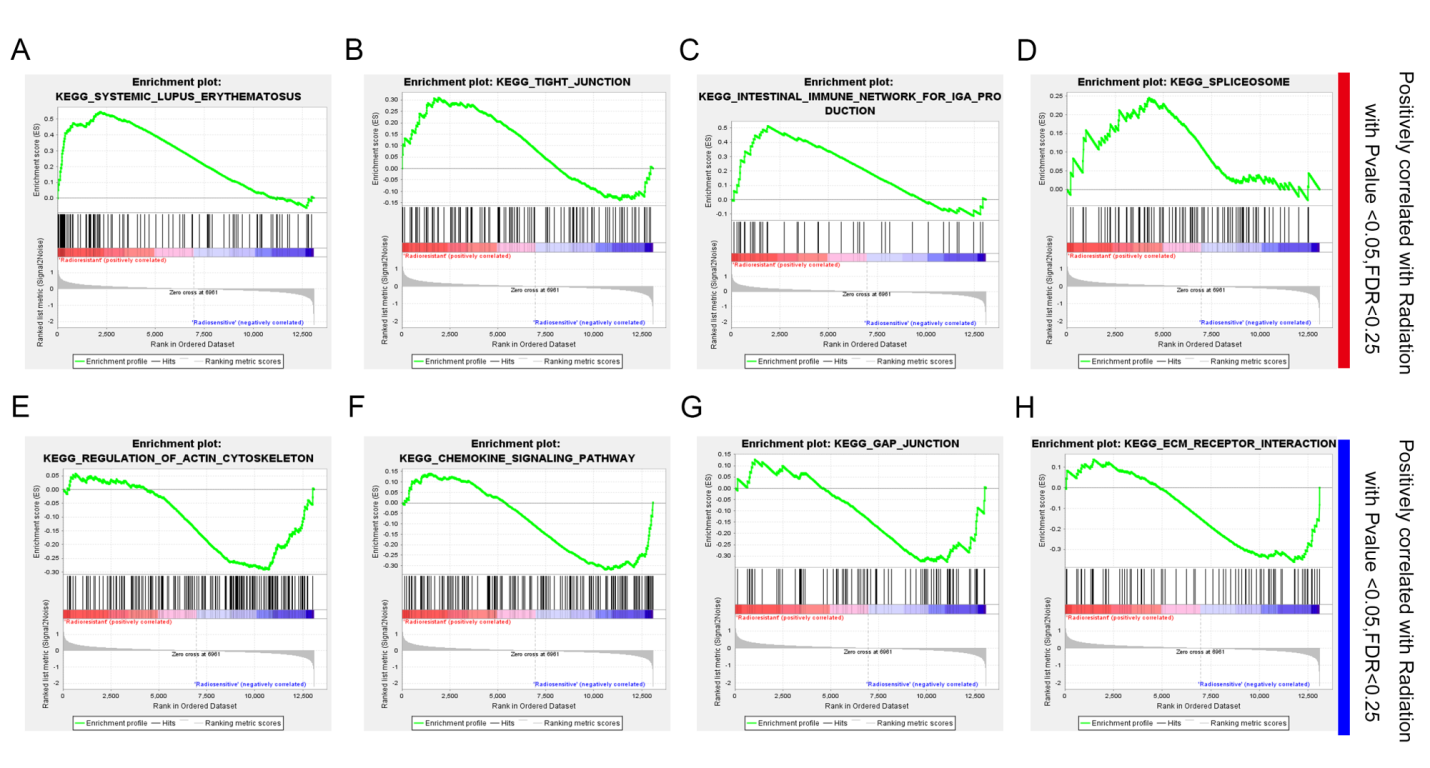

Supplement: Supplemental data [file Suppl_FigureS2.docx]
