# Supplementary material for: Ferroptosis-Related Genes Are Associated with Radioresistance and Immune Suppression in Head and Neck Cancer
Source: Genet Test Mol Biomarkers. 2024 Mar 28;28(3):100–13. doi: 10.1089/gtmb.2023.0193 (PMC10979683; doi:10.1089/gtmb.2023.0193)

**Figure S3. Weighted gene co-expression network analysis (WGCNA).** **(A)** A cluster dendrogram depicts different modules of ferroptosis-correlated DEGs represented by different colors. **(B)** Clustering dendrograms of the genes. The darker the color, the stronger the correlation with clinical traits, including sex, cancer status, story documented, and radiation therapy. **(C)** Samples were clustered to identify outliers. **(D, E)** Distribution of the average gene significance. (F) Scatterplots of gene significance versus module membership.


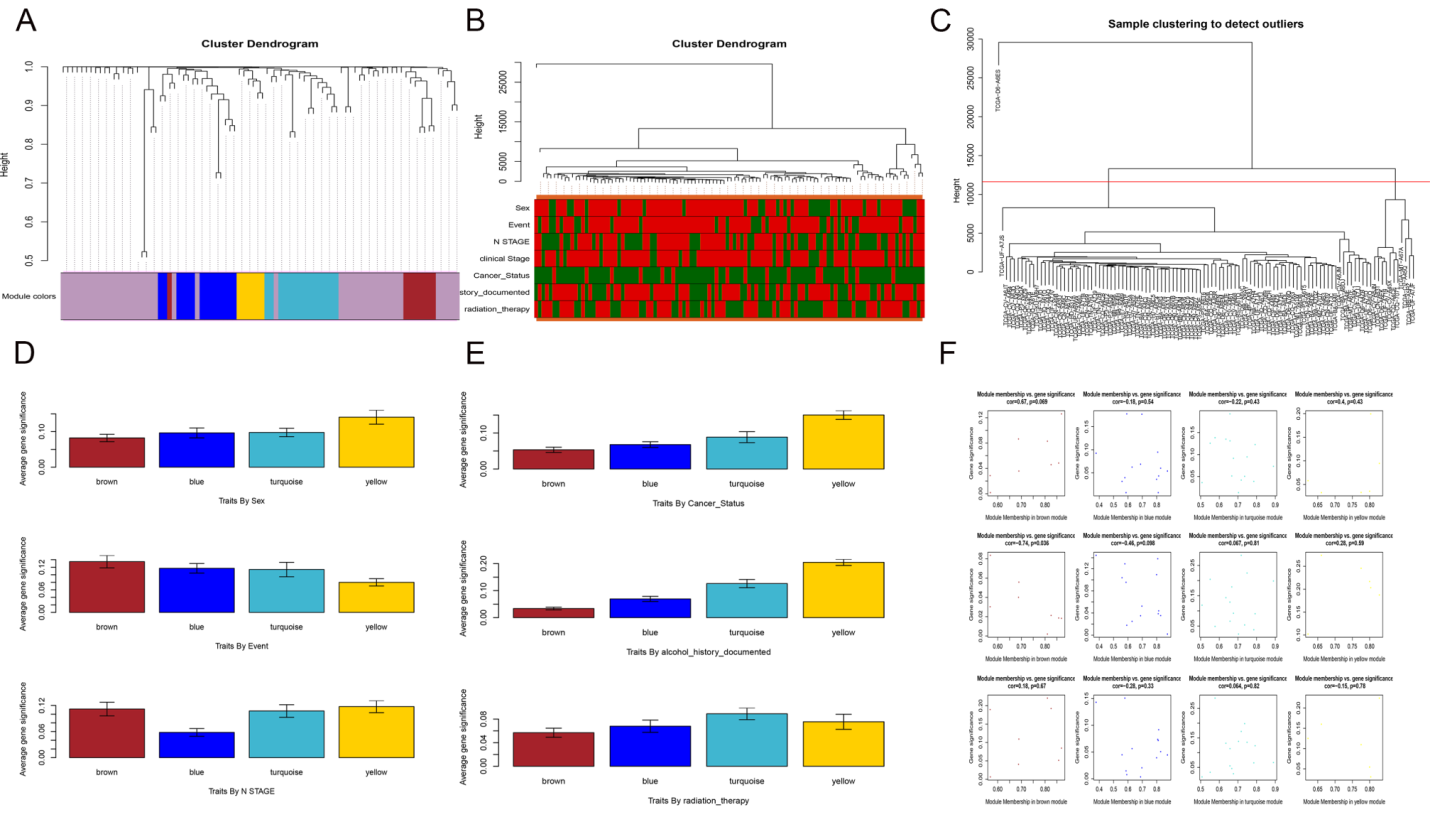

Supplement: Supplemental data [file Suppl_FigureS3.docx]
