# Supplementary material for: Ferroptosis-Related Genes Are Associated with Radioresistance and Immune Suppression in Head and Neck Cancer
Source: Genet Test Mol Biomarkers. 2024 Mar 28;28(3):100–13. doi: 10.1089/gtmb.2023.0193 (PMC10979683; doi:10.1089/gtmb.2023.0193)

**Figure S4. Construction of protein-protein interaction (PPI) network and identification of hub genes.** **(A)** A PPI network was constructed for 107 ferroptosis-correlated DEGs. **(B, C)** Core molecules of the PPI network. (D) Top 25 hub genes.


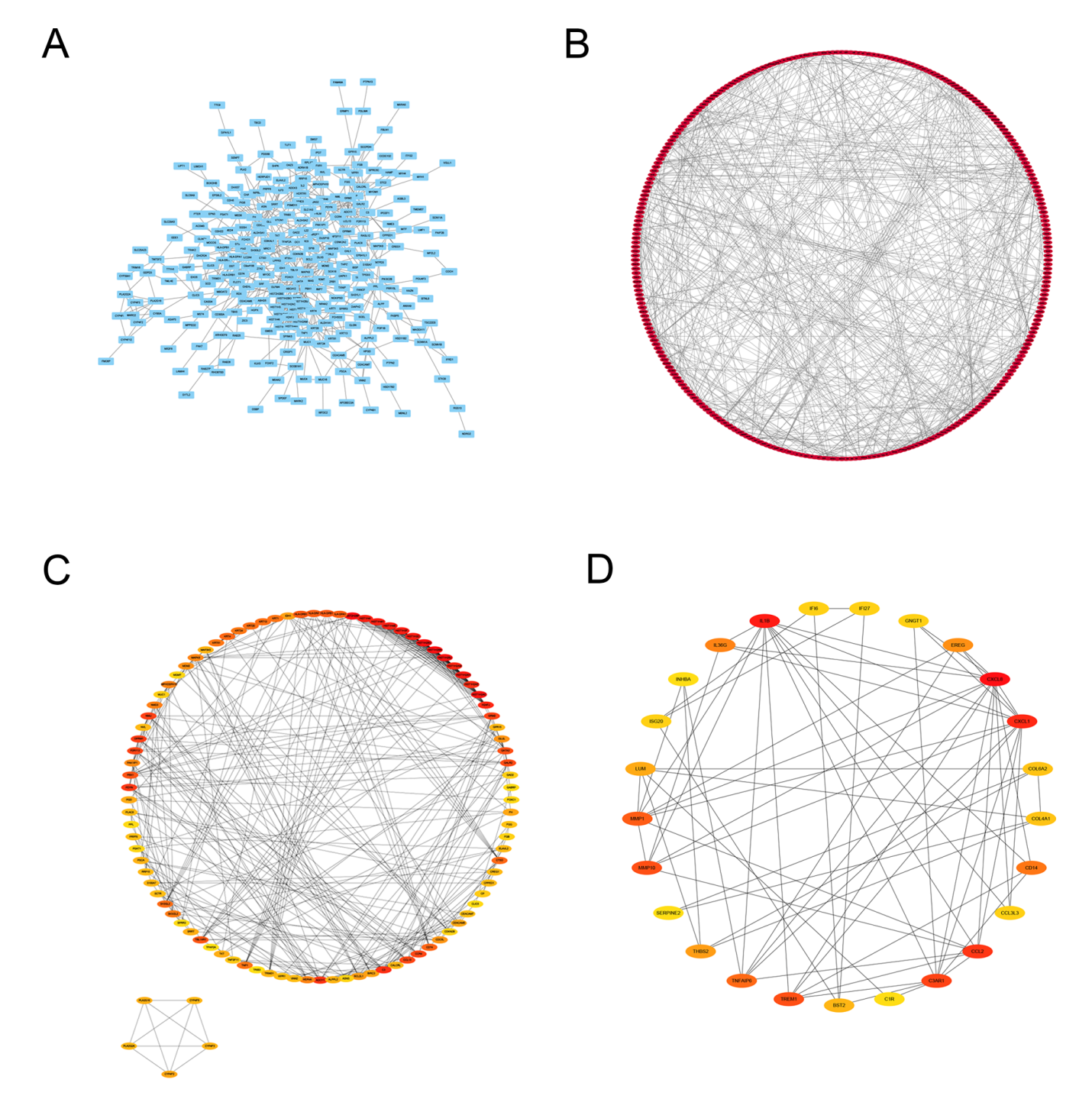

Supplement: Supplemental data [file Suppl_FigureS4.docx]
