# Supplementary material for: Ferroptosis-Related Genes Are Associated with Radioresistance and Immune Suppression in Head and Neck Cancer
Source: Genet Test Mol Biomarkers. 2024 Mar 28;28(3):100–13. doi: 10.1089/gtmb.2023.0193 (PMC10979683; doi:10.1089/gtmb.2023.0193)

**Figure S5. Comparison of hub gene expression between HNC and adjacent normal tissue samples in the TCGA dataset. (A-O)** The mRNA levels of COL6A2 (A), LUM (B), TERM1 (C), TNFAIP6 (D), IL36G (E), IFI6 (F), CCL2 (G), SERPINE2 (H), THBS2 (I), MMP10 (J), IL1B (K), CXCL8 (L), INHBA (M), COL4A1 (N), and BST2 (O) were significantly higher in HNC tissues than in normal adjacent tissues. Data are expressed as the mean ± standard deviation (SD). **P* < 0.05.


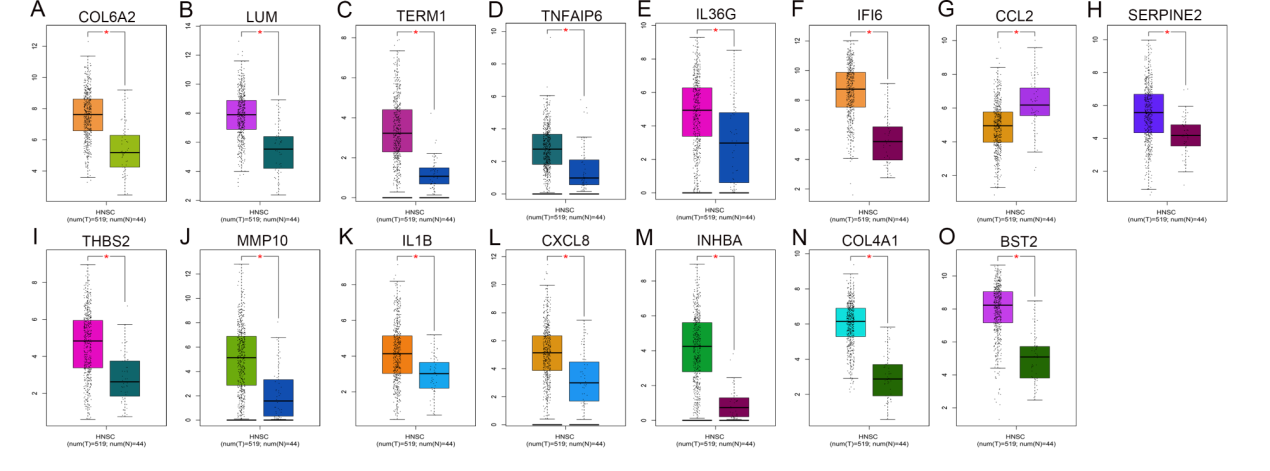

Supplement: Supplemental data [file Suppl_FigureS5.docx]
