# Supplementary material for: Ferroptosis-Related Genes Are Associated with Radioresistance and Immune Suppression in Head and Neck Cancer
Source: Genet Test Mol Biomarkers. 2024 Mar 28;28(3):100–13. doi: 10.1089/gtmb.2023.0193 (PMC10979683; doi:10.1089/gtmb.2023.0193)
Supplement: Supplemental data [file Suppl_TableS7.docx]

**Table S7. GSEA enrichment pathway analysis**.

| NAME | SIZE | ES | NES | NOM p-val | FDR q-val |
| --- | --- | --- | --- | --- | --- |
| KEGG_FOLATE_BIOSYNTHESIS | 11 | 0.68 | 1.47 | 0.04 | 0.20 |
| KEGG_SYSTEMIC_LUPUS_ERYTHEMATOSUS | 99 | 0.55 | 1.45 | 0.00 | 0.00 |
| KEGG_BASAL_TRANSCRIPTION_FACTORS | 30 | 0.50 | 1.44 | 0.05 | 0.00 |
| KEGG_PROPANOATE_METABOLISM | 28 | 0.40 | 1.39 | 0.02 | 0.00 |
| KEGG_ASTHMA | 27 | 0.64 | 1.36 | 0.04 | 0.00 |
| KEGG_SPLICEOSOME | 100 | 0.25 | 1.34 | 0.05 | 0.00 |
| KEGG_AMINO_SUGAR_AND_NUCLEOTIDE_SUGAR_METABOLISM | 37 | 0.40 | 1.34 | 0.15 | 0.00 |
| KEGG_SELENOAMINO_ACID_METABOLISM | 20 | 0.44 | 1.30 | 0.02 | 0.00 |
| KEGG_BETA_ALANINE_METABOLISM | 21 | 0.46 | 1.29 | 0.02 | 0.00 |
| KEGG_THYROID_CANCER | 28 | 0.36 | 1.25 | 0.02 | 0.20 |
| KEGG_BUTANOATE_METABOLISM | 32 | 0.40 | 1.25 | 0.02 | 0.10 |
| KEGG_STEROID_BIOSYNTHESIS | 15 | 0.42 | 1.24 | 0.01 | 0.10 |
| KEGG_TERPENOID_BACKBONE_BIOSYNTHESIS | 14 | 0.46 | 1.18 | 0.04 | 0.25 |
| KEGG_HISTIDINE_METABOLISM | 23 | 0.42 | 1.15 | 0.02 | 0.24 |
| KEGG_AMINOACYL_TRNA_BIOSYNTHESIS | 27 | 0.43 | 1.15 | 0.03 | 0.25 |
| KEGG_ALLOGRAFT_REJECTION | 34 | 0.49 | 1.13 | 0.03 | 0.24 |
| KEGG_INTESTINAL_IMMUNE_NETWORK_FOR_IGA_PRODUCTION | 42 | 0.51 | 1.12 | 0.04 | 0.23 |
| KEGG_OTHER_GLYCAN_DEGRADATION | 12 | 0.47 | 1.12 | 0.03 | 0.12 |
| KEGG_ANTIGEN_PROCESSING_AND_PRESENTATION | 79 | 0.42 | 1.12 | 0.03 | 0.13 |
